# Duck LGP2 Downregulates RIG-I Signaling Pathway-Mediated Innate Immunity Against Tembusu Virus

**DOI:** 10.3389/fimmu.2022.916350

**Published:** 2022-06-15

**Authors:** Tianxu Li, Yanyan Ren, Tingting Zhang, Xinyu Zhai, Xiuyuan Wang, Jinchao Wang, Bin Xing, Runchun Miao, Ning Li, Liangmeng Wei

**Affiliations:** ^1^ Shandong Provincial Key Laboratory of Animal Biotechnology and Disease Control and Prevention, College of Animal Science and Veterinary Medicine, Sino-German Cooperative Research Centre for Zoonosis of Animal Origin of Shandong Province, Shandong Provincial Engineering Technology Research Center of Animal Disease Control and Prevention, Shandong Agricultural University, Tai’an City, China; ^2^ Collaborative Innovation Center for the Origin and Control of Emerging Infectious Diseases, College of Basic Medical Sciences, Shandong First Medical University, Tai’an City, China

**Keywords:** LGP2, RIG-I, signaling pathway, Tembusu virus, innate immunity

## Abstract

In mammals, the retinoic acid-inducible gene I (RIG-I)-like receptors (RLR) has been demonstrated to play a critical role in activating downstream signaling in response to viral RNA. However, its role in ducks’ antiviral innate immunity is less well understood, and how gene-mediated signaling is regulated is unknown. The regulatory role of the duck laboratory of genetics and physiology 2 (duLGP2) in the duck RIG-I (duRIG-I)-mediated antiviral innate immune signaling system was investigated in this study. In duck embryo fibroblast (DEF) cells, overexpression of duLGP2 dramatically reduced duRIG-I-mediated IFN-promotor activity and cytokine expression. In contrast, the knockdown of duLGP2 led to an opposite effect on the duRIG-I-mediated signaling pathway. We demonstrated that duLGP2 suppressed the duRIG-I activation induced by duck Tembusu virus (DTMUV) infection. Intriguingly, when duRIG-I signaling was triggered, duLGP2 enhanced the production of inflammatory cytokines. We further showed that duLGP2 interacts with duRIG-I, and this interaction was intensified during DTMUV infection. In summary, our data suggest that duLGP2 downregulated duRIG-I mediated innate immunity against the Tembusu virus. The findings of this study will help researchers better understand the antiviral innate immune system’s regulatory networks in ducks.

## Introduction

The innate immune system is the first line of defense against infectious pathogens. Pattern-recognition receptors (PRRs) recognize microbial components or structures, known as pathogen-associated molecular patterns (PAMPs), that activate the immune system (1). Typically, viruses produce viral RNA and other PAMPs during the replication process and are recognized by PRRs to activate innate immune responses (2, 3). The effective innate immune response is essential for host survival during viral infection as the virus can also evade or inhibit the immune response in many ways.

PRRs include C-type lectin receptors, Toll-like receptors (TLRs), nucleotide-binding oligomerization domain (NOD)-like receptors (NLRs), and retinoic acid-inducible gene I (RIG-I)-like receptors (RLRs) (4). Among them, RLRs respond to intracellular viral double-stranded RNA (dsRNA) *via* a C-terminal RNA helicase and C-terminal domain (CTD) as a non-self-pattern and then activate downstream signaling (5). The family of RLRs contains three members: (1) RIG-I, (2) differentiation-associated gene 5 (MDA5), and (3) laboratory of genetics and physiology 2 (LGP2), among which RIG-I and MDA5 recognize the viral RNA and form a complex with the mitochondrial antiviral-signaling protein (MAVS) in the cytoplasmic. There are differential roles of RIG-I and MDA5 in RNA virus recognition that have been found. It has been demonstrated that 5’-triphosphate- or 5′-diphosphate-containing RNA structure and small RNA duplexes (influenza A, Newcastle disease, Sendai, vesicular stomatitis, measles, and Hepatitis C viruses) are recognized by RIG-I (6–10).

Duck Tembusu virus (DTMUV) is a plus-strand RNA virus belonging to the Flavivirus genus of the Flaviviridae family, which comprises other arthropod-borne viruses such as Japanese encephalitis virus (JEV) and dengue fever virus (DENV). Early in 2010, an outbreak of DTMUV was observed in Zhejiang and Shanghai in China and rapidly spread throughout the country. DTMUV infection causes nerve malfunction and disruption of the reproductive system, which account for huge losses experienced by duck breeding (11, 12). In addition to infecting ducks of various breeds, the disease can also infect other poultry such as chickens and geese (13) and can cause systemic lesions in infected mice (14), indicating the possibility of cross-host transmission of DTMUV from ducks to other non-avian animals. Importantly, the neutralizing antibodies targeting DTMUV have been isolated from humans (15). Therefore, effective prevention and treatment of the disease are also important public health security. The innate immune response mediated by PRRs plays an important role in the antiviral response, and DTMUV is no exception (16). At present, there are a lot of diagnosis methods for DTMUV, but the antiviral treatment options are few. Therefore, a systematic study of the interaction between DTMUV and the host’s innate immune system will help better to understand the duck’s antiviral innate immune system.

The third member of RLRs, LGP2, was identified in earlier studies and is considered to be the negative regulatory element of the RLR pathway (17). These studies indicated that LGP2 competitively associates with viral RNA to interfere with the recognition by RIG-I and MDA5; in addition, LGP2 forms a complex with MAVS independent of viral infection or dsRNA stimulation, thereby inhibiting the transduction of antiviral signals (17, 18). However, many other studies have shown that LGP2 plays a positive role in regulating the antiviral immune response. Mice with an LGP2 gene deletion cannot effectively produce type I interferon (IFN) during *Picornaviridae* infection. This process occurs because LGP2 can promote RIG-I and MDA5 recognition of viral RNAs through their ATPase domains (19). Previous studies have shown that duRIG-I and duMDA5 recognize the viral RNA of DMTUV and inhibit its replication (20). As an important regulator in the RLRs signaling pathway, duck LGP2 (duLGP2) has been proved to regulate RLRs family members’ duMDA5-dependent anti-DTMUV innate immune responses (21). However, the regulatory role of duLGP2 in duRIG-I-mediated innate immunity against DTMUV infection remains unclear. Previous studies showed that the identity of duLGP2 in humans and mice was only 52.4 and 51.9%, respectively (22). Therefore, it is reasonable to infer that the function of duck LGP2 may be different from that of mammals.

In this study, we provided more detailed experimental data to support the regulatory function of duLGP2 in the duRIG-I-mediated signaling pathway. In addition, we investigated the function of duLGP2 in duRIG-I-mediated anti-DTMUV innate immunity and illustrated that duLGP2 plays a negative role in duRIG-I-mediated limiting DTMUV viral replication and infection. Furthermore, the interaction between duLGP2 and duRIG-I was confirmed by co-immunoprecipitation experiments. This interaction was intensified during DTMUV infection, which inhibits duRIG-I-mediated antiviral innateimmune signaling transduction. These results will advance our knowledge of the biological role of LGP2 in innate immunity and the relationship between LGP2 and innate immunity in ducks.

## Materials and Methods

### Cells and Virus

Duck embryo fibroblast (DEF) cells were prepared by enzyme digestion from 10-day-old duck embryos according to the method described previously (23). The human embryonic kidney 293 (HEK293) cells were kindly provided by Dr. Cheng (Shanghai Jiaotong University). Cells were maintained in Dulbecco’s modified Eagle medium (DMEM, Gibco, Grand Island, NY, USA) containing 10% fetal bovine serum (FBS) (TransGen, Beijing, China), 1% penicillin/streptomycin (Solarbio, Beijing, China), and all incubations were performed in an incubator (5% CO_2_, 37°C). The DTMUV-FX2010 strain used in this study was stored in our laboratory (24). The virus titers were determined by median tissue culture infective dose (TCID_50_) assay in DEF cells using the Reed and Muench calculation (25).

### Plasmids

The expression plasmids pduRIG-I-CARD, pduLGP2-Flag, empty vector pCAGGS, and luciferase reporter promoter plasmid pGL3-chIRF-7-Luc, pGL3-chIFNβ-Luc, pGL3-chNF-κB-Luc, and pTK-Renilla were previously described (21, 26, 27), the pduRIG-I-Flag and pduLGP2-HA plasmids were constructed using Hieff Clone^®^ Plus One Step Cloning Kit (Yeasen, Shanghai, China) in this study. All plasmids were verified by sequencing.

### Cell Transfection, RNA Interference, and Dual−Luciferase Reporter Assay

DEF cells were seeded in 6-well plates incubated overnight to achieve 90%–100% confluence for further transfection. The plasmids (2 μg/well) or siRNAs (100 nM) were transfected into cells using Nulen PlusTrans™ Transfection Reagent (Nulen, Shanghai, China). Three small interfering RNAs (siRNAs) against the duLGP2 and one scramble siRNA as negative control were designed and purchased from GenePharma Co., Ltd (Shanghai, China). The siRNA sequences used were as described in **Table 1**. Knockdown mRNA efficiency was determined for each siRNA by qRT-PCR analysis. For studies involving siRNA and plasmid transfection, cells were transfected with plasmid 24 h after transfection with siRNAs.

**Table 1:** The sequences of siRNAs.

siRNAs	Sequences (5′-3′)	Positions
si-NC (sense)	UUCUCCGAACGUGUCACGUTT	–
si-NC (antisense)	ACGUGACACGUUAGAATT	
si-LGP2-1 (sense)	CCGUCUACAACAAGAUCAUTT	417
si-LGP2-1 (antisense)	AUGAUCUUGUUGUAGACGGTT	
si-LGP2-2 (sense)	CGGACGAUGUUUACUUCUATT	1638
si-LGP2-2 (antisense)	UAGAAGUAAACAUCGUCCGTT	
si-LGP2-3 (sense)	GAGAAGAGGAGGUACAAGATT	1925
si-LGP2-3 (antisense)	UCUUGUACCUCCUCUUCUCTT	

For the reporter gene assays, the cells (24-well plates) were transiently transfected with firefly luciferase reporter (100 ng/well) and pTK-Renilla luciferase reporter (50 ng/well) and indicated expression plasmids or empty vector. After 36 h, luciferase assays were performed as previously described (28) using the Dual-Luciferase Reporter Assay Kit (Vazyme, Nanjing, China). Firefly luciferase activity was normalized to Renilla luciferase.

### Viral Infection

For antiviral effect evaluation, DEF cells were transfected with indicated expression plasmids. After 24 h post-transfection (hpt), the transfected cells were washed twice with PBS and infected with DTMUV-FX2010 (100 TCID_50_) for 1 h, after which the media was changed to low-serum media (2% FBS) after infection. The infected cells were collected for RNA extraction. Viral replication was measured by qRT-PCR described previously (29).

### Co-Immunoprecipitation

For exogenous co-immunoprecipitation experiments, HEK293 cells were seeded in 100-mm dishes (7 × 10^6^ cells/dish) overnight and co-transfected indicated plasmids (10 μg) using a PEI 40K transfection reagent (Servicebio, Wuhan, China). 24h after transfection, HEK293 cells were stimulated with DTMUV or PBS for 2 h, and cells were lysed at 24 hpi in 1 mL Western and IP lysis buffer (25 mM Tris [pH=7.4], 150 mM NaCl, 1% NP-40, 5% glycerol) (New Cell & Molecular Biotech, Suzhou, China) containing with protease inhibitor cocktail (New Cell & Molecular Biotech). Cell debris was removed by centrifugation (12,000 rpm for 15 min at 4°C), and the supernatant was taken as the total protein fraction. 50 μl of supernatant was taken as input samples, and the remaining samples were incubated with 20 μL anti-Flag M2 magnetic beads (Sigma-Aldrich, Louis, MO, USA) for 1 h at room temperature. The immunoprecipitated proteins were analyzed by Western blot assay with the antibodies as indicated.

### Western Blot

The cell lysates were eluted with SDS loading buffer (Beyotime) and boiled for 5 min. Denatured protein samples were separated by 10% SDS-PAGE (New Cell & Molecular Biotech) and transferred to polyvinylidene difluoride (PVDF) membranes (Millipore). Subsequently, the PVDF membranes were blocked in NcmBlot blocking buffer (New Cell & Molecular Biotech) for 30 min at room temperature. Membranes were incubated with mouse anti-Flag mAb (Nulen) (1: 2000), rabbit anti-HA mAb (Cell signaling pathway Danvers, MA, USA) (1: 1000), mouse anti-GAPDH mAb (ABclonal, Wuhan, China) (1: 5000) or mouse anti-β-actin mAb (Abbkine, Wuhan, China) (1: 10000) overnight at 4°C. and then incubated with HRP-conjugated goat anti-mouse or -rabbit IgG antibodies (Abbkine) (1: 10000) for 1 h at room temperature. All membrane washing steps were washed 3 times for 10 min each with 1×TBST at room temperature. Immunoblotting was visualized using the ECL Ultra kit (New Cell & Molecular Biotech).

### qRT-PCR

Total RNA was extracted using the FastPure Cell/Tissue Total RNA Isolation Kit (Vazyme). The resulting RNA was reversely transcribed to cDNA using HiScript III All-in-one RT SuperMix Perfect for qPCR (Vazyme). qRT-PCR was performed using the Hieff UNICON^®^ Universal Blue qPCR SYBR Green Master Mix (Yeasen) on the Roche 96 Light Cycler and under the conditions described previously (23, 30). Primers were synthesized by Tsingke Biotechnology Co., Ltd (Beijing, China), and sequences are shown in **Table 2**. The relative expression of each target gene was analyzed using the 2^−ΔΔCt^ method using duck GAPDH as the internal reference. At least three independent experiments were performed for each sample.

**Table 2:** Primer sequences used in this study.

Primer name	Primer sequence (5′-3′)	Purpose
qLGP2-F	GTGGTGGAGCTGGAGAAGAG	qRT-PCR
qLGP2-R	CCCTGTTCTCCTCAAAGGTG	
qMAVS-F	ACATCCTGAGGAACATGGAC	qRT-PCR
qMAVS-R	AGACCTCCTGCAGCTCTTCG	
qIL-1β-F	TCATCTTCTACCGCCTGGAC	qRT-PCR
qIL-1β-R	GTAGGTGGCGATGTTGACCT	
qIL-2-F	GCCAAGAGCTGACCAACTTC	qRT-PCR
qIL-2-R	ATCGCCCACACTAAGAGCAT	
qIL-6-F	TTCGACGAGGAGAAATGCTT	qRT-PCR
qIL-6-R	CCTTATCGTCGTTGCCAGAT	
qIL-8-F	AAGTTCATCCACCCTAAATC	qRT-PCR
qIL-8-R	GCATCAGAATTGAGCTGAGC	
qIFN-α-F	TCCTCCAACACCTCTTCGAC	qRT-PCR
qIFN-α-R	GGGCTGTAGGTGTGGTTCTG	
qIFN-β-F	AGATGGCTCCCAGCTCTACA	qRT-PCR
qIFN-β-R	AGTGGTTGAGCTGGTTGAGG	
qOAS-F	TCTTCCTCAGCTGCTTCTCC	qRT-PCR
qOAS-R	ACTTCGATGGACTCGCTGTT	
qPKR-F	AATTCCTTGCCTTTTCATTCAA	qRT-PCR
qPKR-R	TTTGTTTTGTGCCATATCTTGG	
qMx-F	TGCTGTCCTTCATGACTTCG	qRT-PCR
qMx-R	GCTTTGCTGAGCCGATTAAC	
qMHC-I-F	GAAGGAAGAGACTTCATTGCCTTG	qRT-PCR
qMHC-I-R	CTCTCCTCTCCAGTACGTCCTTCC	
qMHC-II-F	CCACCTTTACCAGCTTCGAG	qRT-PCR
qMHC-II-R	CCGTTCTTCATCCAGGTGAT	
qGAPDH-F	ATGTTCGTGATGGGTGTGAA	qRT-PCR
qGAPDH-R	CTGTCTTCGTGTGTGGCTGT	
qDTMUV-F	CGCTGAGATGGAGGATTATGG	qRT-PCR
qDTMUV-R	ACTGATTGTTTGGTGGCGTG	

F, forward primer; R, reverse primer; q, qRT-PCR.

### Statistical Analyses

Expression levels and endogenous housekeeping gene, GAPDH, were analyzed using the 2^−ΔΔCt^ method (31). All experimental data were presented as mean ± standard error of the mean (SEM). Student’s t- and one-way analysis of variance (ANOVA) tests were used to determine the statistical significance of the differences using GraphPad Prism 8.0.1 software (GraphPad Software Inc., SanDiego, CA). *P* < 0.05 was considered to be statistically significant, *P* < 0.01 was highly significant, and *P* < 0.001 was extremely significant.

## Results

### pduRIG-I-Flag and pduLGP2-Flag Expression Plasmids are Expressed in DEF Cells

**Figure 1:**
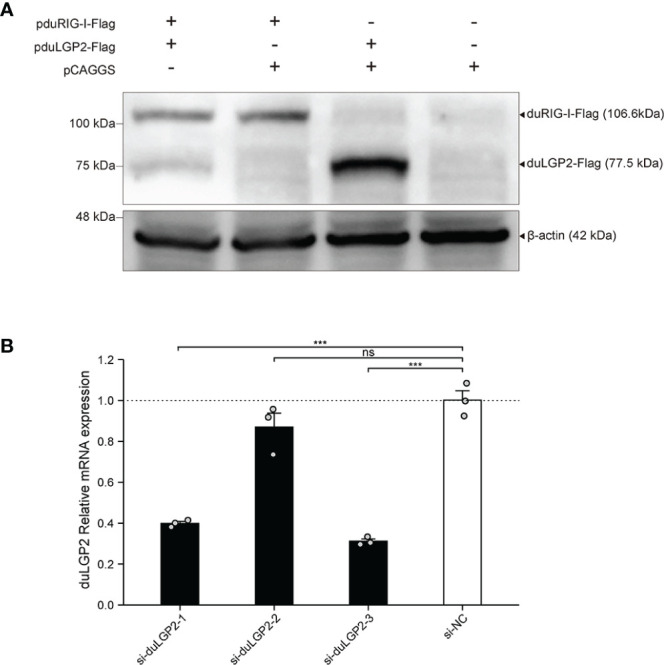
Expression of pduLGP2-Flag and pduRIG-I-Flag, and Knockdown of duLGP2 by siRNAs in DEF cells. **(A)** DEF cells were transiently transfected with the expression plasmids or empty plasmids pCAGGS. Western blot was performed at 36 hpt. **(B)** DEF cells were transiently transfected with siRNAs. The duLGP2 relatively mRNA expression was detected at 36 hpt using qRT-PCR. All samples were analyzed in triplicate, and all data were expressed as means ± SEM. ***Extremely significant (*P* < 0.001); ns, no significant difference.

To detect the expression pduRIG-I-Flag and pduLGP2-Flag, the DEF cells were co-transfected with indicated expression plasmids. The duLGP2 and duRIG-I expression levels were detected by Western blot. As shown in **Figure 1A**, Flag-tag duRIG-I and duLGP2 plasmids were coexpressed in DEF cells.

### siRNA Knock Downed of Endogenous duLGP2 in DEF Cells

To knockdown endogenous duLGP2 in DEF cells, each siRNA was transfected into DEF cells, and knockdown efficiency was assessed by qRT-PCR in 24 hpt. The results showed that si-diLGP2-1 and si-duLGP2-3 had a pronounced effect on duLGP2 mRNA expression (*P* < 0.01) (**Figure 1B**). However, the si-duLGP2-3 with better knockdown efficiency (68.8%, *P* < 0.01) were used for the following experiments.

### DuLGP2 Involved in the Regulation of duRIG-I-Mediated IFN-β Signaling Pathway

**Figure 2:**
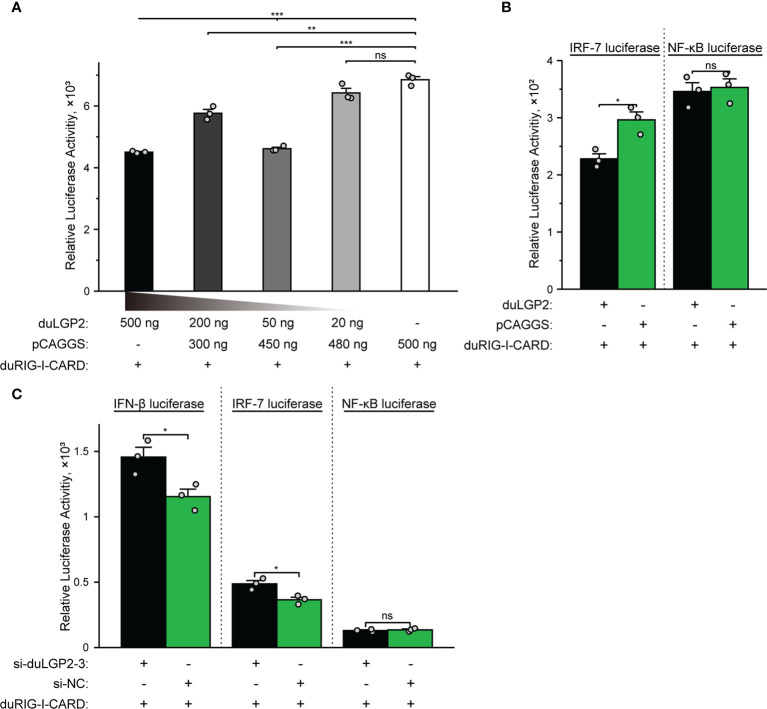
duLGP2 regulates the duRIG-I-mediated IFN-β signaling pathway. **(A)** DEF cells were transiently co-transfected with the IFN-β promoter construct and indicated expression plasmids or empty plasmids pCAGGS. **(B)** DEF cells were transiently co-transfected with plasmids IRF-7 or NF-κB promoter construct with indicated expression plasmids or empty plasmids pCAGGS. **(C)** DEF cells were transiently transfected with siduLGP2-3 or si-NC, and the cells were transfected with indicated plasmids 24 h after transfection with siRNAs. Cell samples were lysed at 24 hpt, and luciferase activities were quantified by normalization with Renilla luciferase activity. All samples were analyzed in triplicate, and all data were expressed as means ± SEM. *Significant difference (*P* < 0.05); **Highly significant difference (*P* < 0.01); ***Extremely significant (*P* < 0.001); ns, no significant difference.

The structure of full-length RIG-I without RNA ligand showed that RIG-I is in a signaling-repressed in mammals (32). Therefore, we choose pduRIG-I-CARD to detect the regulation role of duLGP2 on duRIG-I signaling. LGP2 protein has been proved to inhibit RIG-I-mediated IFN-β, IRF-3/7, and NF-κB promoter activities in mammalian cells (33). DEF cells were co-transfected with indicated expression plasmids, firefly luciferase reporter gene, and internal reference plasmids to investigate whether duLGP2 regulates duRIG-I-mediated IFN-β expression. The results demonstrated that overexpression of duLGP2 derepresses the duRIG-I-mediated IFN-β promoter activity in a dose-dependent manner (**Figure 2A**). Moreover, duLGP2 may inhibit duRIG-I-mediated IFN-β transcription *via* IRF-7 rather than the NF-κB signaling pathway (**Figure 2B**). In contrast, knockdown of duLGP2 led to an opposite effect on the IFN-β and IRF-7 signaling pathway, and there were still no significant changes for NF-κB (**Figure 2C**).

### DuLGP2 Regulates the Expression of duRIG-I-Mediated Cytokines

**Figure 3:**
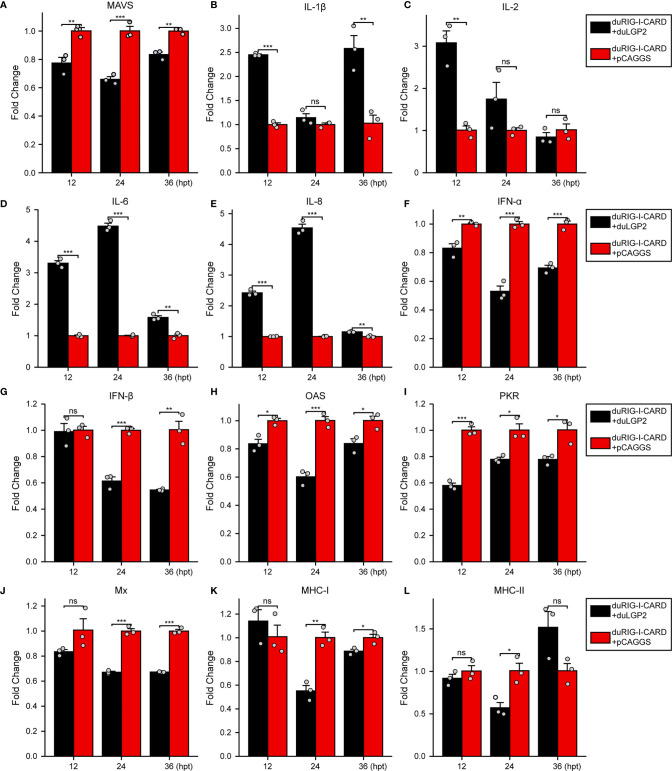
Overexpression of duLGP2 regulates duRIG-I-mediated cytokines expression. DEF cells were transiently co-transfected with indicated expression plasmids or empty plasmids pCAGGS. Cell samples were collected for analysis of cytokine detection at different time points. **(A)** MAVS **(B)** IL-1β **(C)** IL-2 **(D)** IL-6 **(E)** IL-8 **(F)** IFN-α **(G)** IFN- β **(H)** OAS **(I)** PKR **(J)** Mx. **(K)** MHC-I **(L)** MHC-II. The relative expression of gene mRNA was calculated using the 2^−ΔΔCt^ method with GAPDH serving as a normalization gene and mean control values as a baseline reference, and the values in the control groups (pCAGGS co-transfection) were set to 1. All samples were analyzed in triplicate, and all data were expressed as means ± SEM. *Significant difference (*P* < 0.05); **Highly significant difference (*P* < 0.01); ***Extremely significant (*P* < 0.001); ns, no significant difference.

**Figure 4:**
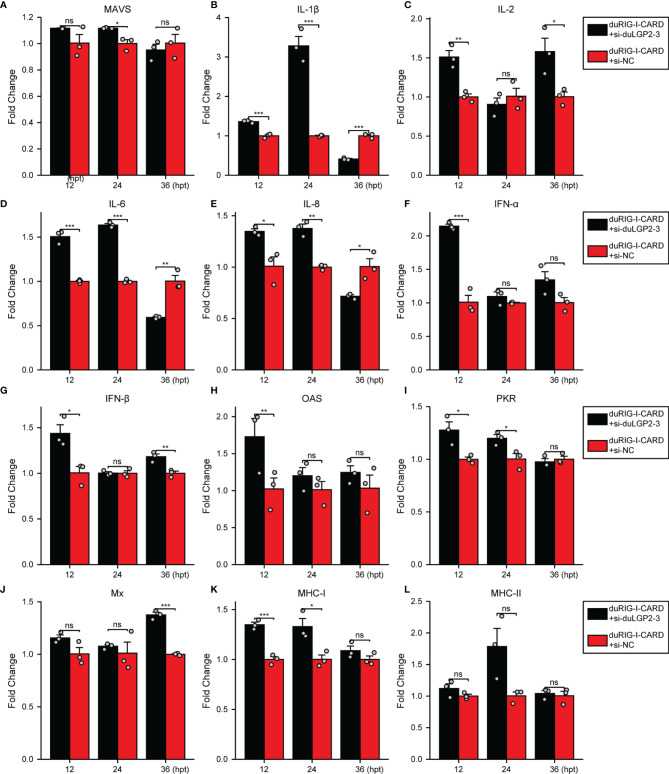
Knockdown of duLGP2 regulates duRIG-I-mediated cytokines expression. DEF cells were transiently transfected with siduLGP2-3 or si-NC, and the cells were transfected with indicated plasmids or empty plasmids pCAGGS 24 h after transfection with siRNAs. Cell samples were collected for analysis of cytokine detection at different time points. **(A)** MAVS **(B)** IL-1β **(C)** IL-2 **(D)** IL-6 **(E)** IL-8 **(F)** IFN-α **(G)** IFN- β **(H)** OAS **(I)** PKR **(J)** Mx. **(K)** MHC-I **(L)** MHC-II. The relative expression of gene mRNA was calculated using the 2^−ΔΔCt^ method with GAPDH serving as a normalization gene and mean control values as a baseline reference, and the values in the control groups (pCAGGS co-transfection) were set to 1. All samples were analyzed in triplicate, and all data were expressed as means ± SEM. *Significant difference (*P* < 0.05); **Highly significant difference (*P* < 0.01); ***Extremely significant (*P* < 0.001); ns, no significant difference.

To further examine the regulatory role of duLGP2 on the duRIG-I signaling pathway, DEF cells were co-transfected with indicated expression plasmids, and the duRIG-I signaling-related cytokines mRNA expression levels were detected by qRT-PCR. The results showed that duRIG-I-mediated MAVS, type I IFNs, ISGs and MHCs were all significantly down-regulated by duLGP2 overexpression (**Figures 3A, F–L**), and the expression of duRIG-I-mediated proinflammatory cytokines were upregulated by duLGP2 (**Figures 3B–E**). In contrast, knockdown of duLGP2 led to an opposite effect on the duRIG-I-mediated type I IFNs, ISGs, and MHC-I (**Figures 4F, G–K**), and although the expression of duRIG-I-mediated proinflammatory cytokines (IL-1β, -6 and -8) was upregulated by knockdown of duLGP2 at 12-24 hpt, but down-regulated at 36 hpt (**Figures 4B–E**).

### DuLGP2 Promotes DTMUV Replication in DEF Cells Overexpressing duRIG-I

**Figure 5:**
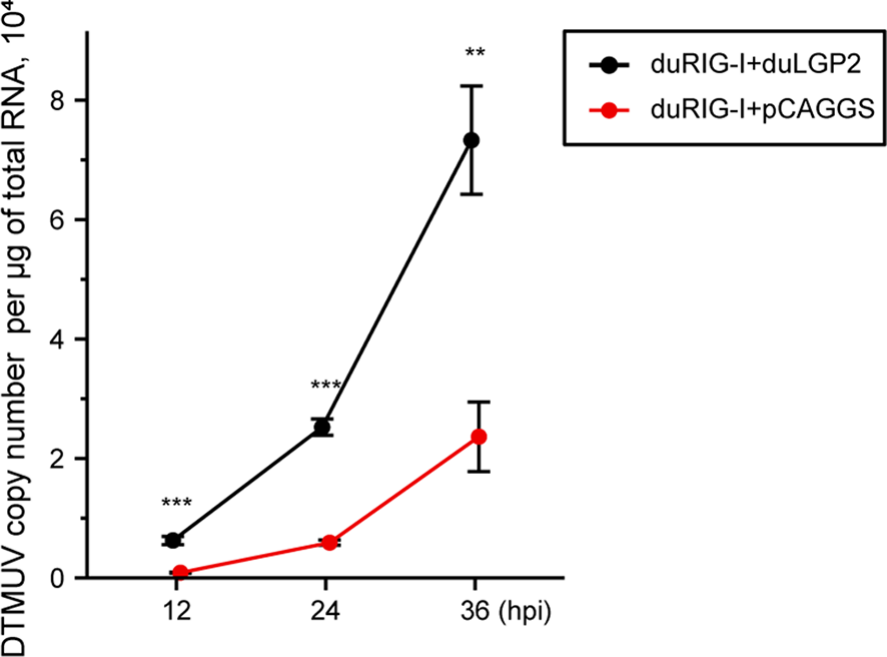
duLGP2 inhibits duRIG-I-mediated anti-DTMUV activity. Cells were transiently co-transfected with indicated expression plasmids or empty plasmids pCAGGS. Cells were infected with DTMUV at 24 hpt and collected for analysis of virus replication at different time points. All samples were analyzed in triplicate, and all data were expressed as means ± SEM. **Highly significant difference (*P* < 0.01); ***Extremely significant (*P* < 0.001).

Previously, duRIG-I has been proved to inhibit DTMUV replication *in vitro* (34, 35). To examine whether duLGP2 can regulate RIG-I-mediated anti-DTMUV ability, the co-overexpressing duRIG-I and duLGP2 or empty vector pCAGGS DEF cells were inoculated with DTMUV-FX2010. The results showed that viral titers of duLGP2-overexpressing DEF cells were higher than those of the control cells at all the tested time points, and it was upregulated by 7.26- (*P* < 0.001), 4.29- (*P* < 0.001), and 3.10-fold (*P* < 0.01), at 12, 24, and 36 hpi, respectively (**Figure 5**).

### DuLGP2 Inhibits duRIG-I Mediated IFN-β Signaling Pathway During DTMUV Infection

**Figure 6:**
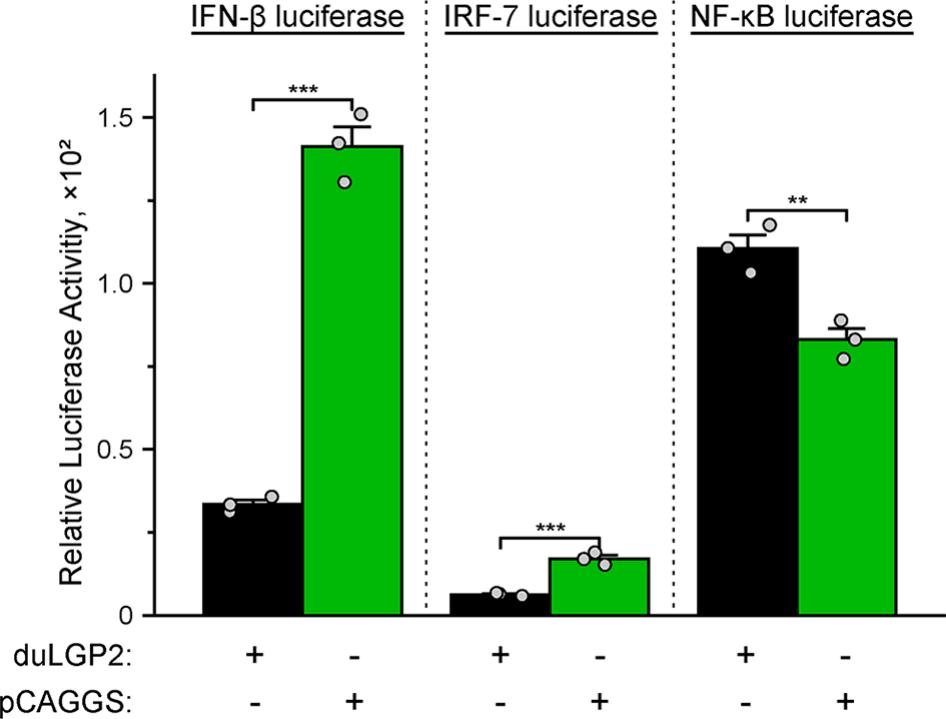
Overexpression of duLGP2 regulates the duRIG-I-mediated IFN-β signaling pathway during DTMUV infection. DEF cells were transiently co-transfected with IFN-β, IRF-7, or NF-κB promoter construct with indicated expression plasmids or empty plasmids pCAGGS. Cells were infected with DTMUV at 24 hpt and lysed at 12 hpi, and luciferase activities were quantified by normalization with Renilla luciferase activity. All samples were analyzed in triplicate, and all data were expressed as means ± SEM. **Highly significant difference (*P* < 0.01); ***Extremely significant (*P* < 0.001).

To investigate whether duLGP2 regulates duRIG-I-mediated IFN-β expression during DTMUV infection. DEF cells were co-transfected with indicated expression and reporter gene plasmids. The results showed that duLGP2 led to significant down-regulation of duRIG-I-mediated IFN-β promoter activity during DTMUV infection, and this regulatory effect is likely exerted *via* the IRF-7 signaling pathway. However, the duRIG-I-mediated NF-κB activity displayed a degree of up-regulation (**Figure 6**).

### DuLGP2 Regulates the Expression of duRIG-I Mediated Cytokines During DTMUV Infection

**Figure 7:**
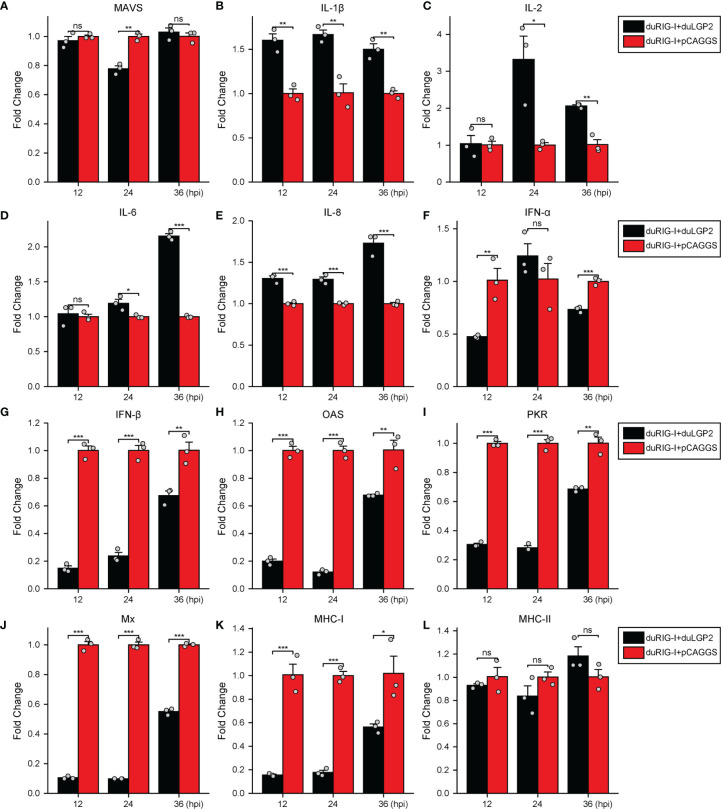
duLGP2 regulates duRIG-I-mediated cytokine expression during DTMUV infection. DEF cells were transiently co-transfected with indicated expression plasmids or empty plasmids pCAGGS. Cells were infected with DTMUV at 24 hpt and collected for analysis of cytokine detection at different time points. **(A)** MAVS **(B)** IL-1β **(C)** IL-2 **(D)** IL-6 **(E)** IL-8 **(F)** IFN-α **(G)** IFN- β **(H)** OAS **(I)** PKR **(J)** Mx. **(K)** MHC-I **(L)** MHC-II. The relative expression of gene mRNA was calculated using the 2^−ΔΔCt^ method with GAPDH serving as a normalization gene and mean control values as a baseline reference, and the values in the control groups (pCAGGS co-transfection) were set to 1. All samples were analyzed in triplicate, and all data were expressed as means ± SEM. *Significant difference (*P* < 0.05); **Highly significant difference (*P* < 0.01); ***Extremely significant (*P* < 0.001); ns, no significant difference.

To further examine the regulatory role of duLGP2 on the duRIG-I signaling pathway during DTMUV infection. DEF cells were co-transfected with indicated expression plasmids. After 24 hpt, the cells were infected with DTMUV-FX2010, and the duRIG-I signaling-related cytokines expression levels were detected by qRT-PCR. As expected, the results indicated that the RIG-I downstream key adaptor protein MAVS, type I IFNs (IFN-α and -β), ISGs (PKR, OAS, and Mx), and MHC-I were significantly down-regulated by duLGP2 during DTMUV infection (**Figures 7A, F–K**). In addition, duLGP2 upregulated the expression of duRIG-I mediated several key pro-inflammatory cytokines during DTMUV infection, including IL-1β, -2, -6, and -8 (**Figures 7B–E**).

### DuLGP2 Interacts With duRIG-I

**Figure 8:**
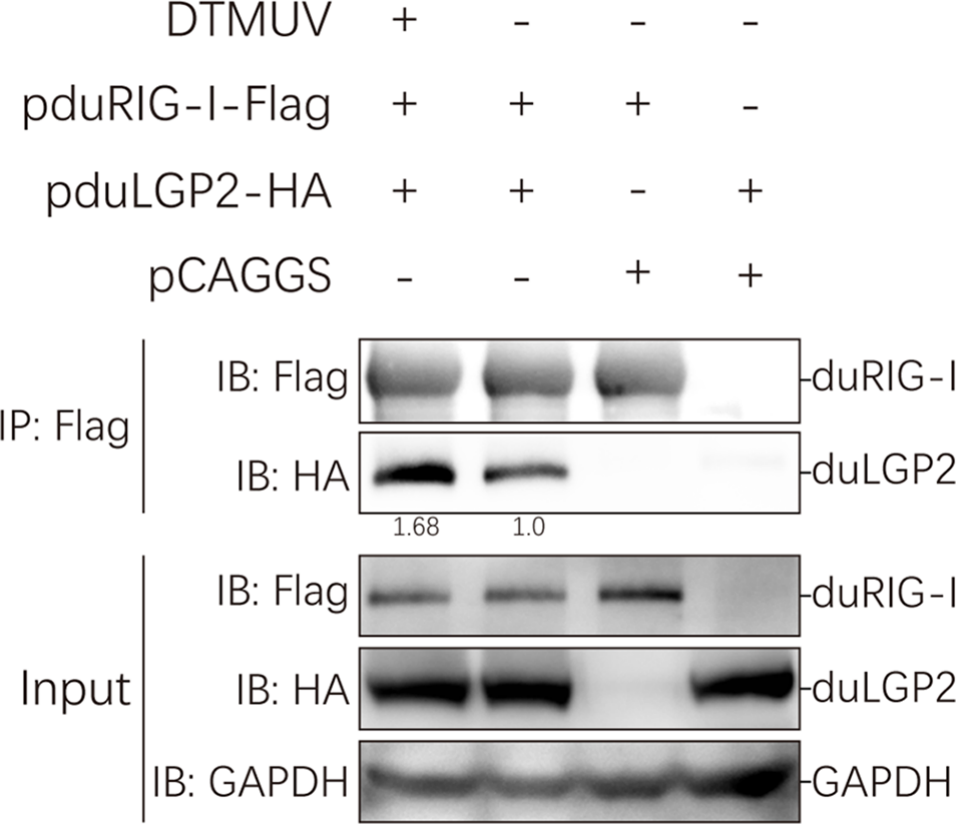
duLGP2 interacts with duRIG-I. HEK 293 cells were transiently co-transfected with indicated expression plasmids or empty plasmids pCAGGS. Cells were infected with DTMUV or PBS at 24 hpt and lysed at 24 hpi. Immunoprecipitates were analyzed by Western blot with the indicated antibodies, and the relative gray values of the bands were calculated using the ImageJ software.

Our study indicated that duLGP2 is a negatively regulatory molecule functioning of the duRIG-I-mediated signaling pathway. However, the mechanisms involved in this regulation are currently unknown. Reciprocal co-immunoprecipitation assays were performed to determine if duLGP2 regulates duRIG-I through interaction between the proteins. As shown in **Figure 8**, when the lysates were immunoprecipitated with anti-Flag-tag magnetic beads, duLGP2 could be detected *via* an immunoblotting assay using an anti-HA antibody in immunoprecipitated protein complexes. The result suggested an interaction between duRIG-I and duLGP2, and this interaction was intensified during DTMUV infection.

## Discussion

RLRs have been identified as major PRRs that respond to viral RNA in the cytoplasm (5). RIG-I, a member of the RLRs family, recognizes RNA structures of multiple viruses, including DTMUV RNA, which is activated and binds to adaptor protein MAVS, leading to NF-κB and IRF-3/7 pathway activation (34, 36). LGP2, the third member of the RLRs family, lacks the CARD domains compared with RIG-I and MDA5 and has been proven a regulatory factor of the RIG-I and MDA5 signaling pathway (36, 37), and the regulatory function of LGP2 in RLRs-mediated signaling pathways is controversial. Previous studies have also proven that duLGP2 has high homology with mammals (22), and it is speculated that the function of duLGP2 may be similar to that in mammals. A recent study from our group demonstrated that duLGP2 plays a negative role in duMDA5-dependent anti-DTMUV innate immune responses (21). However, the regulatory effect of duLGP2 on duRIG-I-mediated anti-DTMUV infection is currently not known.

It is well established that type I IFN plays an important role in antiviral immunity. Previous studies have shown that IRF-7 and NF-κB are key transcriptional regulators that activate type I IFN and inflammatory cytokines ducks (27, 38). The present experiment indicates that the duRIG-I-mediated IFN-β promoter activity was effectively down-regulated by co-transfection with duLGP2, and this effect may be the result of inhibition of IRF-7 rather than an NF-κB dependent signaling pathway (**Figure 2**). Furthermore, the qRT-PCR results showed that the duRIG-I-mediated expression level of adaptor protein MAVS, pro-inflammatory cytokines, Type I IFNs, and ISGs was significantly down-regulated by overexpression duLGP2 (**Figure 3**), and these results have been validated by knockdown of duLGP2 endogenous expression (**Figures 2**, **4**). It remains controversial how LGP2 regulates RIG-I function (19, 32, 37, 39–41). The present study indicated that duLGP2 might function as a negative regulator in duRIG-I signaling.

DTMUV is a viral disease that is currently very serious to the duck industry. Both duRIG-I and duMDA5 participate in the anti-virus immune response, and duRIG-I has a more pronounced anti-DTMUV effect than duMDA5 (34). RIG-I is a well-known strong inducer of the type I IFN response (3). Moreover, the expression of RIG-I is positively regulated by IFNs, IL-1β, and LPS (42–47). An intense inflammatory reaction is induced by DTMUV infection, which may lead to the host’s death (48). Since the duRIG-I expression is strongly induced in response to DTMUV infection, it is likely to play an important role in host defense (49–53). We speculate that the duRIG-I-mediated immune response during DTMUV infection may be one of the reasons for this strong inflammatory response. Consequently, it is important to understand the duRIG-I-mediated anti-DTMUV immune function regulation.

Studies of knockout mice have shown that the loss of LGP2 is highly susceptible to encephalomyocarditis virus infection (19). Additionally, the effect of porcine RIG-I against RNA virus infection was positively regulated by LGP2. In contrast, another study showed that LGP2 binds to protein kinase activator A (PACT) to blocks of RIG-I-mediated IFN-β promoter signaling (54). In the absence of RNA ligands, the CARDs fold back to the C-terminal portion of RIG-I, which causes it to be in an auto-inhibited state (55). Upon recognizing the viral RNA of flavivirus, the RIG-I undergoes a conformational change to trigger type I IFN signaling (56, 57). Multiple studies demonstrate that DTMUV can impair the host’s innate immune response *via* non-structural proteins resulting in viral escape (58–60). A recent study showed that duck interferon-induced protein 35 (IFI35) binds to duRIG-I to counteract its antiviral signal, the interaction enhanced by DTMUV infection (61), which indicated that the virus evades host immunity by several mechanisms. The present study suggests that duRIG-I-mediated IRF-7 signaling was down-regulated by duLGP2 during DTMUV infection, leading to the down-regulation of IFN-β promoter activity (**Figure 6**). Additionally, the mRNA expression of cytokines was detected during DTMUV infection. As expected, the results showed that duRIG-I-mediated type I IFNs (IFN-α and -β) and downstream ISGs (OAS, PKR, and Mx) were found to be significantly down-regulated in the duLGP2 overexpression group during DTMUV infection (**Figure 7**). Type I IFNs was considered the most important cytokine involved in the antiviral immune response, which directly and/or indirectly drives antiviral effects through the induction of other mediators and induces the activation of immune cells (62). These results collectively indicate that the anti-DTMUV antiviral capability of duRIG-I was down-regulated by duLGP2, which ultimately resulted in enhanced DTMUV replication *in vitro* (**Figure 5**). Additionally, we demonstrate that regulation of the duLGP2 is achieved by direct binding of the duRIG-I, and this interaction was intensified during DTMUV infection (**Figure 8**).

Interestingly, duRIG-I-CARD- or full-length duRIG-I- (DTMUV infection) mediated pro-inflammatory cytokines (IL-1β, IL-2, IL-6, IL-8) expression level was significantly promoted by overexpression of duLGP2 (**Figures 2**, **7**). Recently, DTMUV has been proven to cross the blood-brain barrier into the central nervous system and cause nonsuppurative encephalitis in ducklings. The blood-brain barrier will eventually be destroyed by the virus, thus triggering a subsequent “inflammatory storm”. There is no doubt that RLRs play an important role in DTMUV infection-mediated inflammatory response (49, 63). The present experiment shows that duLGP2 is involved in the expression of inflammatory factors in the duRIG-I-mediated anti-DTMUV immune response and the regulatory role of duLGP2 on the duRIG-I may *via* NF-κB signaling pathway and contribute to promoting inflammation and regulating inflammatory responses (**Figure 6**).

In conclusion, our study provides further evidence for the regulatory role of duLGP2 in duRIG-I-mediated anti-DTMUV. Although LGP2 per se function impacts RLRs has been controversial, we demonstrated that duLGP2 negatively affects duRIG-I-mediated anti-DTMUV immune responses.

## Data Availability Statement

The original contributions presented in the study are included in the article/supplementary material. Further inquiries can be directed to the corresponding author.

## Ethics Statement

The animal study was reviewed and approved by Shandong Agricultural University Animal Care and Use Committee (SDAUA-2020-005).

## Author Contributions

TL wrote the manuscript and performed most of the experiments. YR, TZ, XZ, XW, JW, BX, and RM experimented and wrote the discussion. NL analyzed the data. LW designed the study and reviewed the manuscript. All authors read and approved the final manuscript.

## Funding

This work was supported by the National Natural Science Foundation of China (31972664) and the Local Science and Technology Development Fund Project Guided by the Central Government of Shandong Province (YDZX20203700004857).

## Conflict of Interest

The authors declare that the research was conducted in the absence of any commercial or financial relationships that could be construed as a potential conflict of interest.

## Publisher’s Note

All claims expressed in this article are solely those of the authors and do not necessarily represent those of their affiliated organizations, or those of the publisher, the editors and the reviewers. Any product that may be evaluated in this article, or claim that may be made by its manufacturer, is not guaranteed or endorsed by the publisher.
